# How can we use social network analysis to better understand Chimpanzee and Gorilla sociality and communication?

**DOI:** 10.1007/s10071-025-01980-z

**Published:** 2025-07-14

**Authors:** Anna Ilona Roberts, Sam George Bradley Roberts

**Affiliations:** 1 McDonald Institute for Archaeological Research, University of Cambridge, 13A Fitzwilliam Street, Cambridge, CB2 1QH UK; 2 School of Natural Sciences and Psychology, Liverpool John Moores University, Byrom Street, Liverpool, L3 3AF UK

**Keywords:** Social brain hypothesis, Communicative roots of complex sociality and cognition, Social network analysis, Chimpanzee, Gorilla, Group size, Brain size, Stress, Communication, Sociality

## Abstract

An important element in understanding the evolution of human sociality is to understand the factors that governed the evolution of social organisation in our closest living relatives. The ‘social brain hypothesis’ proposes that the complex social world of primates is especially cognitively demanding, and that this imposed intense selection pressure for increasingly large brains. Group size in primates is strongly correlated with brain size but exactly what makes larger groups more ‘socially complex’ than smaller groups is still poorly understood. Chimpanzees and Gorillas are among our closest living relatives and they exhibit remarkable diversity in various aspects of their social organisation both within and across species. They are thus excellent species in which to investigate patterns of sociality and social complexity in primates, and to inform models of human social evolution. We propose a program of research that will provide the first systematic insight into how social structure differs in small, medium and large groups of Chimpanzees and Gorillas, to explore what makes larger groups more socially complex than smaller groups. Further, we propose to investigate how these variations in social structure in different size groups are affected by the social organisation of the species. Chimpanzees live in a fluid fission-fusion social system, whereas Gorillas have more stable, cohesive groups. To carry out both the within and between species comparisons, we advocate use of social network analysis, which provides a novel way to describe and compare social structure. This program of research will therefore lead to a new, systematic way of comparing social complexity across species, something that is lacking in current comparative studies of social structure. Considering that hominins were likely characterized by a fission-fusion social structure, comparing the social complexity of such systems with that of more stable groups may yield valuable insights into the evolution of human sociality.

## Introduction

An important element in understanding the evolution of human sociality is to understand the factors that governed the evolution of social organisation in our closest living relatives. Primate sociality is often described as particularly complex, and primates have relatively large brains for their body size compared to other mammals. According to the ‘social brain hypothesis’, the cognitive demands of navigating complex social environments have driven the evolution of larger brains in primates (Dunbar and Shultz 2007b). There is a strong correlation between brain size—particularly the neocortex—and group size, with species that form larger social groups typically exhibiting a higher neocortex-to-brain ratio. Nonetheless, why larger groups are more socially complex than smaller ones remains insufficiently understood. Chimpanzees and Gorillas are among our closest living relatives and they exhibit remarkable diversity in various aspects of their social organisation both within and across species. They are thus excellent model species to investigate patterns of sociality and social complexity in primates, and to inform models of human social evolution.

The purpose of this paper is to review research in a newly emerging field of social and communicative complexity of primates and identify key areas for future research. First, we examine how social structure differs in small, medium and large groups of Chimpanzees and Gorillas to explore what makes larger groups more socially complex than smaller groups. Second, we explore how these variations in social structure in different size groups are affected by the social organisation of the species. Chimpanzees are characterised by a fluid fission-fusion social system, whereby community membership is stable, but party membership varies spatially and temporally (Goodall 1986). In contrast Gorillas have more stable, cohesive groups, whereby membership of the group is stable both spatially and temporally (Doran and McNeilage 1998). Social network analysis provides a novel way to describe and compare social relationships and social structure (Koyama et al. 2017; Krause et al. 2009; Sueur et al. 2011). Examining these links will therefore lead to a novel, systematic way of comparing social structure and social complexity in humans, primates and other animals, something that is sorely lacking in current comparative studies of social structure. Given a fission-fusion system is likely to have characterised hominins, a comparison of the social complexity involved in fission-fusion and more stable social systems will provide new insights into human social evolution (Aureli et al. 2008a, b; Foley and Gamble 2009).

A defining characteristic of primate social systems is their high degree of complexity. Evidence for this is found in the strong association between neocortex size and typical group size, suggesting that evolutionary pressures favoured an expanded neocortex to support the cognitive demands of managing social information. According to the social brain hypothesis, cognitive capacity—as indicated by relative neocortex volume—constrains the number of individuals with whom an animal can maintain cohesive social ties. Rather than interacting uniformly with all group members, primates typically invest in enduring and differentiated social bonds, often extending to both kin and non-kin.

Grooming represents a key strategy through which primates sustain social bonds, and can comprise up to 20% of their daily activity budget. Empirical evidence indicates a positive correlation between grooming time and group size, suggesting that individuals in larger groups must invest more time in maintaining social bonds compared to those in smaller groups (Lehmann et al. 2007). Nevertheless, the time available for social interaction is inherently constrained by competing demands such as foraging, resting, and locomotion. Consequently, primate group size appears to be limited by two distinct factors: the size of the neocortex, which determines the maximum number of social relationships an individual can cognitively manage, and the availability of time for grooming, which is essential for sustaining the cohesion required to prevent group fragmentation. As group size increases, individuals face growing challenges in sustaining social bonds with all members, which can lead to reduced group cohesion and eventual fission. In baboons, for instance, the likelihood of group splitting rises with larger group sizes. This phenomenon appears to result not from ecological factors such as foraging inefficiency or heightened predation risk, but rather from constraints on the time available for maintaining social relationships, limiting individuals’ capacity to invest adequately in social interactions (Henzi et al. 1997).

Group size is often used as a proxy for social complexity, primarily because the number of possible dyadic and triadic interactions increases exponentially with group size. However, this metric remains a relatively coarse indicator and does not adequately explain the factors that render larger groups more socially complex than smaller ones. It also overlooks how the internal structure of a group influences the quantity and nature of social relationships that individuals must cognitively manage. Moreover, the precise aspects of sociality and relationship maintenance that impose significant demands on neural processing capacity remain poorly understood. The social brain hypothesis itself is based on the relationship between *social complexity* (i.e. managing a more complex network of relationships) and neocortex size, not simply on the quantitative relationship between group size and brain size. Primates possessing relatively larger neocortices tend to exhibit increased rates of social play, employ more sophisticated male mating strategies, demonstrate greater use of tactical deception, show a higher propensity for coalition formation, and display elevated levels of social learning. Whilst this suggests that primates with larger neocortices do display a higher level of ‘social complexity’ in their behaviour, what is lacking is a systematic and detailed comparison of how group size affects individual relationships and social structure. Further, how social structure varies with group size is likely to be affected by the social system of the species in question.

A key dimension along which primate social systems vary is the degree of temporal stability in spatial cohesion. In species exhibiting fission–fusion social dynamics, overall group structure is fluid, with subunits forming and dissolving in response to factors such as activity type (e.g., feeding or resting) and the spatial distribution of resources (Aureli et al. 2008a, b). The term “fission–fusion dynamics” captures the variability in group cohesion and individual association patterns over time. Gorillas exhibit low fission–fusion dynamics, characterized by stable group membership and high spatial cohesion, such that individuals typically encounter all other group members on a daily basis (Doran and McNeilage 1998; Robbins and Robbins 2018). The majority of Gorilla groups consist of one adult male (although up to four males may be present in a group) and a number of unrelated females, plus juveniles and infants. The mean group size is 9, with a range of 2 to 34. The groups are spatially and temporally cohesive. Further, the strongest bonds within the groups are between the adult females and the silverback. Gorillas are folivores, and because they rely on an abundant, easily available food resource, there is little competition between groups and home ranges are typically small, between 3 and 15 km^2^ (Doran and McNeilage 1998; Robbins and Robbins 2018).

In contrast, Chimpanzees exhibit a high degree of fission–fusion dynamics (Goodall 1986; Lehmann and Boesch 2004). They belong to communities within which individuals associate in temporary subgroups, or “parties,” that fluctuate in size, composition, and duration. The community size can range from 20 to 150, and the community as a whole is rarely seen together in one place (Goodall 1986; Lehmann and Boesch 2004; Reynolds 2005a). Chimpanzees are frugivores and communities defend a communal home range, which is typically much larger than that of Gorillas, ranging from 5 to 35 km^2^. As a result, individuals within the broader community may encounter one another only sporadically, sometimes with intervals of several weeks between interactions. Nevertheless, they are able to recognize their community members and sustain long-term social relationships despite these periods of separation (Goodall 1986; Reynolds 2005b).

Thus Chimpanzees (frugivores with a fluid fission-fusion system) and Gorillas (folivores with stable, cohesive groups) are at opposite ends of a continuum of ape dietary and social patterns. A comparison of Gorillas and Chimpanzees therefore offers an ideal opportunity to examine both how the patterns of association between individuals changes with increasing group size, and how the underlying social structure affects these changes in patterns of association. An increase in group size among Gorillas primarily leads to more frequent daily encounters with a greater number of individuals. In contrast, an increase in Chimpanzee community size imposes greater cognitive demands, as individuals must monitor a larger network of indirect social relationships, including those interactions where affiliation occurs only infrequently. How Gorillas and Chimpanzees adjust their social strategies and patterns of association in groups of differing sizes is thus informative of the key cognitive and time-budget pressures involved in sociality (Aureli and Schino 2019a; Freeberg et al. 2012).

As well as furthering our understanding of primate sociality, understanding the social structure of systems with varying degrees of fission-fusion dynamics is of crucial importance for understanding the course of human social evolution (Foley and Gamble 2009). Fission–fusion social dynamics are a defining feature of both Chimpanzee and bonobo societies and are also commonly observed among contemporary hunter-gatherer populations. This pattern supports the inference that such dynamics were likely present in the social organization of the last common ancestor shared by Chimpanzees, bonobos, and modern humans (Aureli et al. 2008a, b; Foley and Gamble 2009). Moreover, human evolutionary history is marked by a consistent increase in brain size, which is thought to have been accompanied by a parallel expansion in typical social group size (Aiello and Dunbar 1993). Thus understanding the complexity involved in fission-fusion systems, as compared to more stable social groups, and how this complexity changes in groups of different sizes, will help us understand the social evolution in our hominin ancestors (Dunbar et al. 2014; Foley and Gamble 2009).

Understanding how social complexity varies with group size and the degree of fission–fusion dynamics necessitates a systematic and comparative framework for defining and quantifying social complexity across groups and species. At present, no universally accepted metric exists for this purpose, and the development of a standardized measure applicable across taxa has been referred to as the “grail of social analysis” (Whitehead 2008, p. 20). In this paper, we propose the use of social network analysis to develop such a quantitative measure that can be applied across a wide number of primate and non-primate species. A network represents a system consisting of individual components, referred to as ‘nodes,’ and the relationships or interactions between them, known as ‘edges.’ Recent advances in computing power, in mathematics and statistical physics and in the availability of large-scale electronic databases have resulted in new paradigms for the characterisation of the structure of complex networks in a range of fields, including electrical power grids, transport systems, the world wide web and metabolic reaction networks (Watts 2004). There is also an increasing realisation that network analysis - by providing common techniques and modes of analysis - can lead to a greater synthesis across the many disciplines in the mathematical, biological and social sciences in which network-related problems arise.

In social networks analysis, each node usually represents an individual, and each edge (or, as used in this proposal, ‘tie’) represents some measured social interaction or association (e.g. time spent grooming). The social network approach is grounded in the notion that the patterning of ties in which individuals are embedded has important consequences for these individuals. Network analysis provides a way of exploring how the patterning of individual social relationships builds up to produce the complex social structure observed at the group or population level. Understanding this link between individual behaviour and population-level phenomena is a long standing challenge in ecology and evolutionary biology (Croft et al. 2007). Network theory provides novel insights into the properties of social structure in groups that are not possible either by considering the interactions between pairs of individuals in isolation, or by studying the average properties of the group as a whole (Croft et al. 2007; Wey et al. 2008).

Further, recently developed methods for identifying natural subgroups in networks provide a way to assess intermediate-level groupings, defined as groups of individuals that associate with each other more than with other individuals in the network. These structures may be especially difficult to detect in fission-fusion systems where group membership is unstable over time and space. Thus, using network analysis, subgroups of Chimpanzees that preferentially associate with each other could be identified within the larger Chimpanzee community, revealing the internal structure of the community in a way that would not be possible purely based on individual relationships or association indices.

Network analysis therefore provides a well-developed and established set of definitions and quantitative measures (based on explicit mathematical formulae) for objectively characterising both individual relationships and social groups. As many of the measures can be standardized by dividing by group size, systematic comparisons between different groups and species can be made (Sundaresan et al. 2007). Using these quantitative measures of relationships, statistical models about social relationships and social structure can be tested (Wey et al. 2008). By comparing networks both within and between species, network methods help to determine the extent to which social structure is driven by ecology or phylogeny (Sundaresan et al. 2007).

## Specific background

To date most of the studies which examined primate social complexity and cognitive ability have used the approach of comparing the neocortex ratio to group size, or the neocortex ratio to behaviours thought to be indicative of social complexity such as tactical deception, complex male mating strategies or social play (Dunbar and Shultz 2007b). However, group size is a relatively crude measure of social complexity, and does not provide a detailed explanation of why larger groups are more complex than smaller ones, or of how the way in which the group is structured affects the number and types of relationships an individual primate has to keep track of. Further, examining how individual behaviours are related to the neocortex ratio is a piecemeal approach, and only focuses on a limited number of the many behavioural interactions that go into forming complex social relationships.

In order to assess how social complexity varies across groups of different sizes, and with different levels of fission-fusion dynamics, a systematic way of defining, measuring and comparing social complexity across different groups and species is required. Currently, there is a lack of such a standardized measure of social complexity. In this paper we propose use of social network analysis to explore in detail how the patterning of social relationships varies between small, medium and large groups of Gorillas and Chimpanzees both *within* species, and *between* species. In smaller social groups, primates are typically able to maintain strong, multifaceted relationships with most or all group members, supported by frequent interactions involving behaviours such as grooming, vocalisations, gestures, and spatial proximity. However, with increasing group size, the social bonds primates have with group members will weaken, and there will be less frequent interaction and an increasing dissociation between different types of behaviours, as animals use different behaviours to maintain the different types of ties. These weak, indirect ties are cognitively complex to manage, and this is especially true in species living in fission-fusion social systems, where the frequency of social interactions between two individuals is typically much lower than in stable groups (Barrett et al. 2003). Thus, in larger groups one may predict that there will be increasing dissociation between networks based on different measures of behaviour (e.g. grooming, vocalisations, gestures, proximity), as primates use different behaviours to maintain ties of different strengths. Possibly, there will be an increased repertoire of both vocal and gestural communication because of the need to use increasingly sophisticated strategies to maintain an increasing number of differentiated ties. Finally, it could be predicted that the structuring of the group may differ, with an increasing number of sub-groups forming in larger groups. Thus, for example, a large community of Chimpanzees may in fact consist of a number of distinct sub-groups only loosely tied together. However, to date this relationship between the complexity of social behaviour and group size has not been examined systematically.

The complexity of a social system arises from the complexity of individual relationships among its members, as the broader social structure emerges from these underlying, fine-scale interactions. Thus, to examine why larger groups are more complex than smaller groups, it is necessary to analyse what happens to the patterning of these individual relationships as group size increases. Understanding social complexity in primates requires detailed understanding of the ways individuals interact to establish and sustain relationships over time, as these interactions underpin the socially complex nature of primate life. While other species, such as wildebeest and buffalo, may gather in large groups and show high levels of spatiotemporal cohesion, these tend to be fluid associations lacking stable membership and enduring individual bonds. In these species, spatiotemporal cohesion depends on factors such as predation risk, and animals disperse once proximity to others is no longer necessary (Dunbar 2024). In contrast, primates typically live in groups with consistent membership and form enduring social ties with specific group members. In primates, spatiotemporal cohesion is often dependent on the strength of social bonds rather than global pressures such as predation risk (Dunbar 2024). Thus, variations in social structure (e.g. the extent of differentiation in the strength of ties within the group) will influence and will be influenced by the degree of spatiotemporal cohesion within the groups. These social relationships can have direct consequences for fitness; for instance, in baboons, female sociality—measured through behaviours such as grooming and spatial proximity—is positively correlated with offspring survival (Silk 2007). The dynamic, multifaceted quality of these relationships, along with the cognitive demands of managing both dyadic and third-party social connections, is thought to drive the complexity of primate social life.

To maintain these complex social bonds, primates use many different types of behavioural interactions. It is well established that primates use grooming to maintain their social relationships. The amount of time primates devote to grooming increases with group size, suggesting that individuals in larger groups must invest more time in maintaining social bonds (Lehmann et al. 2007). This extra grooming time appears to be invested in strengthening the social bonds with existing social partners, rather than investing their grooming time into strengthening their ties with all group members (Dunbar 2024). Nevertheless, vocal and gestural communication also play a vital role in managing social relationships among primates. Despite their importance, the function of vocalisations—particularly gestures—in sustaining social ties has received comparatively less attention than grooming, even though these modalities hold significant potential for advancing our understanding of the evolution of human language. While time limitations restrict the extent to which grooming can be used (Lehmann et al. 2007), vocal and gestural signals are less bound by such constraints and may therefore provide an efficient means of regulating social interactions as group size increases (McComb and Semple 2005). Additionally, the size of primate vocal repertoires correlates with group size, indicating that vocal communication may support larger group sizes within primate species (McComb and Semple 2005). A similar relationship between group size and vocal repertoire complexity has also been noted in chickadee birds (Freeberg et al. 2012). A key challenge for the study of primate sociality is thus evaluating the relative importance of grooming, vocalisations and gestures in the maintenance of primate social networks (Seyfarth and Cheney 1993), and exploring how primates in groups of increasing size use these behaviours differentially to maintain their social relationships. In a complex social system, individuals may need to use a variety of different behavioural interactions (grooming, vocalisations, gestures, proximity, visual attention, coalitionary support) to manage social relationships, whereas in less complex social systems individuals would use fewer types of behavioural interactions to manage their relationships (Lehmann and Dunbar 2009a). The extent to which networks based on these different types of behavioural interactions overlap can be statistically tested, providing a quantitative measure of the extent to which primates use different types of behaviours to maintain their relationships, and the extent to which this varies with group size and social organisation (Lehmann and Dunbar 2009b). This could be used as a measure of social complexity that can be applied across a wide number of primate and non-primate species.

Group living is generally contingent upon use of social knowledge to predict outcomes of social interactions, but the capacity to retain and manipulate social information is inherently limited (Dunbar 2024). Research shows that in larger groups, subgroups form because the cognitive effort of tracking social relationships causes stress that naturally leads to group fragmentation, and hence loss of the benefits that group-living provides (Causse et al. 2022; Dunbar 2024). When the cognitive effort of tracking multiple social relationships causes maladaptive stress, the reward value of processing social information diminishes, prompting individuals to withdraw from actively processing and updating information about social relationships (Bogdanov et al. 2021; Garbarino and Edell 1997; Roberts et al. 2022a, b; Shany-Ur et al. 2014). Evidence shows that stress diminishes behavioural and brain responses to expectancy violations, leading to a shift to a reliance on habitual processing when predicting others’ future behaviour (Cracco et al. 2020; Lenow et al. 2014). Specifically, stress increases the tendency to predict another’s future actions based on past behaviour, rather than current goals (Witt et al. 2023). As a result, animals react to conspecifics in a stimulus driven way, rather than integrating social information from wide range of sources to update their knowledge of social interactions. For instance, stressed baboons reduce their number of grooming partners, and focus their grooming on their few key allies (Crockford et al. 2008). This reduction in sociality stems not from the fact that primates are overwhelmed by tracking of environmental states (e.g. location of predators or prey) or phenological states (e.g. timing of fruiting), but directly due to tracking of behavioural and mental states, which arguably is a more complex and fluctuating component of primates life (Dunbar 1998, 2024).

**Fig. 1:**
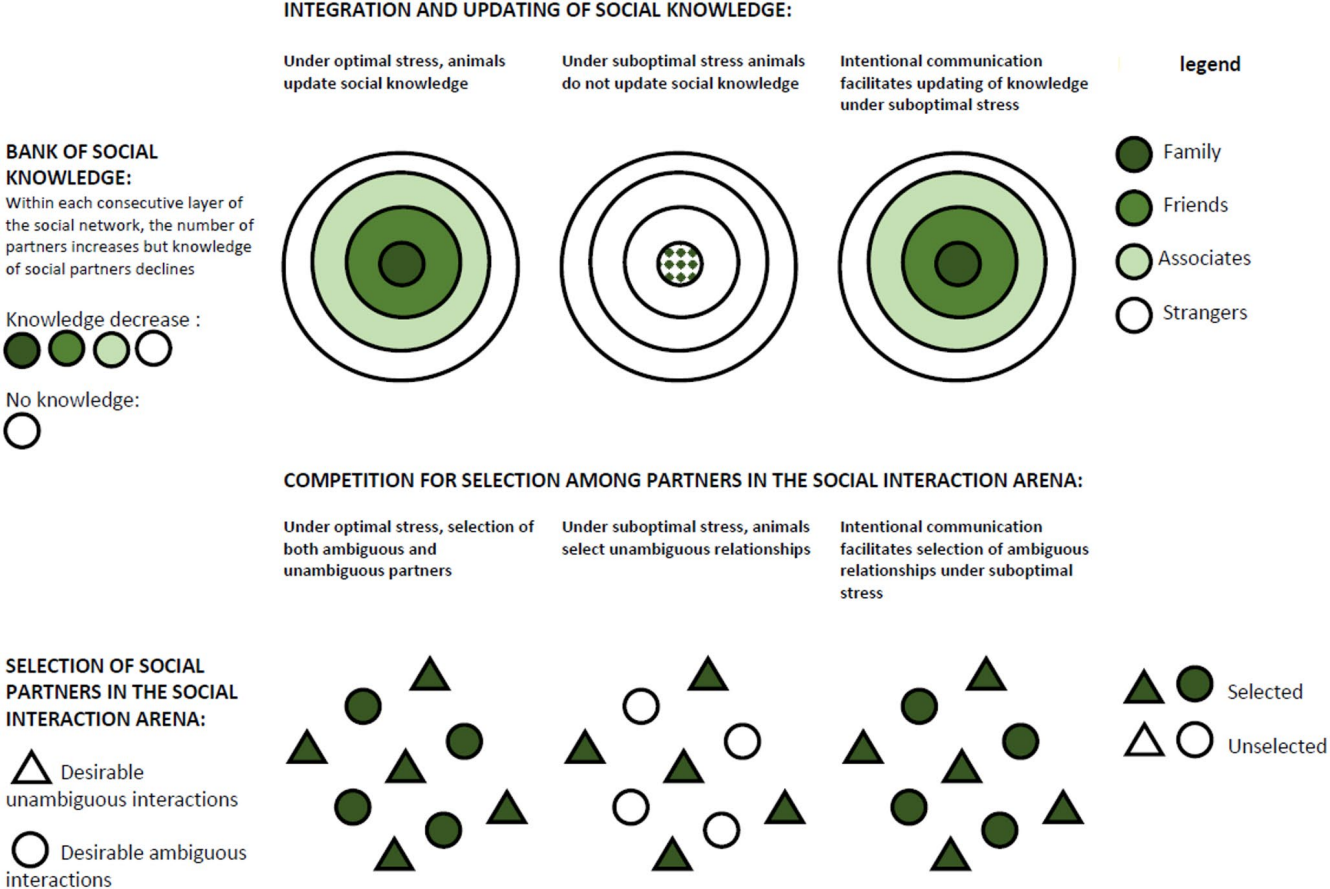
The Communicative Roots of Complex Sociality and Cognition Hypothesis. This hypothesis explains the relationship between communicative and social complexity (Roberts and Roberts 2020, 2022; Roberts et al. 2022a, b). Tracking of numerous social relationships in large social groups leads to stress, which depletes the ‘bank of social knowledge’. because the reward value of processing social information diminishes. Specifically, stress diminishes behavioural and brain responses to expectancy violations, leading to a reliance toward habitual processing when predicting others’ future behaviour. This manifests as a lack of motivation to integrate information and update social knowledge of conspecifics. Intentional communication increases the relevance of social interactions and motivates animals to integrate information to update their social knowledge of conspecifics. Animals adjust the number of strong and weak social bonds they maintain to reduce the cognitive demands of managing social relationships. This leads to the ‘social interaction arena’ being differentiated and more complex, allowing the social cohesion of complex social groups to be maintained

Under conditions of high uncertainty and formation of subgroups, primates are expected to adjust social differentiation to reduce the cognitive demands behind tracking social relationships to a level that can sustain group cohesion and stability (Roberts and Roberts 2022; S. G. Roberts et al. 2022a, b) (Fig. 1). Social differentiation arises from flexible adjustment of the number of strong and weak bonds animals have and thus the amount of information that has to be cognitively managed. Primates track and retain information about individuals with whom they share close social bonds, while information about less familiar group members is less well remembered. Typically, primates store detailed social information for only a small number of individuals—often not exceeding five conspecifics (Escribano et al. 2022; Mac Carron et al. 2016). The ability of the primate to retain and process information is dependent on their ability to allocate memory by selectively focusing on relevant information (Noudoost and Moore 2011). Intentional communication (indexed by the presence of audience checking, response waiting or elaboration) plays an important role in this process because it increases the relevance of social interaction to the recipient (Roberts and Roberts 2022; Roberts et al. 2022a, b).

**Fig. 2:**
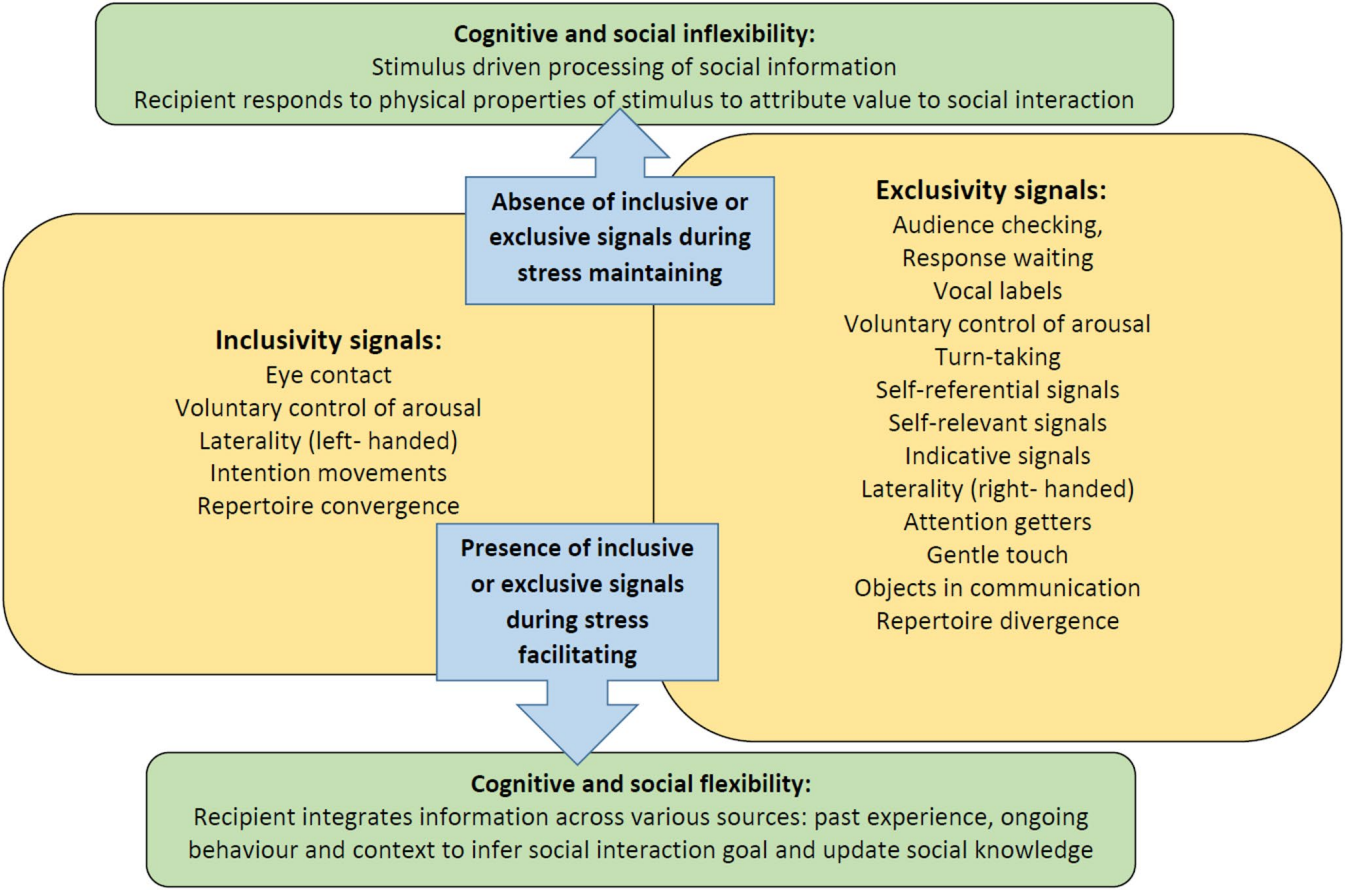
Examples of inclusive and exclusive communication in primates. The cognitive demands of tracking social information in large social groups lead to stress and stimulus driven processing of social information, whereby primates do not integrate and update their knowledge of social relationships. This leads to inaccurate predictions of the outcomes of social interactions and thus overall group instability. The Hypothesis for Communicative Roots of Complex Sociality and Cognition posits that intentional communication (indexed by the presence of audience checking, response waiting or elaboration) facilitates updating of social knowledge and therefore promotes accurate predictions of outcomes of social interactions (Damjanovic et al. 2022; Roberts 2024; Roberts and Roberts 2020, 2022, 2025; Roberts et al. 2022a, b). Intentional communication achieves this objective by increasing the relevance of the social interaction to the recipient of signalling, who then integrates social knowledge (Roberts et al. 2022a, b). However intentional signalling is differentiated according to the inclusivity or exclusivity of the social relationship to account for the differences in a number of strong and weak social bonds in social groups in different social systems, group sizes and social positions in the network

In groups where many animals compete for attention, intentional communication motivates animals to integrate their social knowledge and update their understanding of the outcomes of social interactions because relevance is enhanced (Corbetta et al. 2008; Patel et al. 2019; Roberts 2024; Roberts and Roberts 2020, 2022, 2025; Roberts et al. 2022a, b). However, as social systems vary in the number of strong and weak ties, communication strategies are differentiated. In large groups of Chimpanzees, group members have a small number of strong social bonds and many weak social bonds. Thus, maintaining social cohesion in Chimpanzees focuses on broadening the number of strong social connections by enhancing inclusivity. In contrast, in large groups of Gorillas, group members have many strong social bonds, and a fewer number of weak social bonds. Thus, in Gorillas, strengthening the smaller number of social connections by increasing exclusivity maintains social cohesion. For instance, inclusive communication of moderate quality (i.e. forms of signals that are commonly used, signalling similarity between signaller and recipient) would map onto group identities that are more trusting of strangers, whereby individuals forge social bonds they can depend on for support (Fig. 2). In contrast, exclusive communication of very high quality (i.e. distinctive forms of signals, rare in use or signalling dissimilarity with surrounding audience) would be characteristic of forms of social ranking that create or enhance exclusivity (Roberts and Roberts 2017) (Fig. 2). Identifying this role of intentional signals in social differentiation provides a promising basis for understanding how the communicative complexity of primates is related to within and between group variation in social complexity (Roberts and Roberts 2022, 2025).

### Specific objectives

We propose to use network analysis to examine how the patterning of social relationships varies with group size in Gorillas and Chimpanzees. Specifically:

#### To explore how social relationships vary between small, medium and large groups *within* species

Living in large groups is thought to be more cognitively demanding than living in smaller groups. However, there is little understanding of what it is about large groups that makes them socially complex. It is important to examine how network structure varies with group size in Gorillas and Chimpanzees in order to explore and quantify this social complexity.

#### To explore how group size affects social relationships *between* species

How group size affects social relationships is likely to be affected by the social organisation of the species. By comparing the patterns of social relationships in small, medium and large groups of Chimpanzees and Gorillas, the influence of social organisation (fission-fusion vs. stable groups) on the level of social complexity individual animals have to deal with can be explored. Given a fission-fusion system is likely to have characterised hominins, a comparison of the social complexity involved in fission-fusion and more stable social systems will provide new insights into human social evolution.

Social network analysis provides an excellent way to objectively characterise the patterning of social relations, but does not provide a comprehensive understanding of the proximate mechanisms involved in regulating social relationships. Thus, as well as undertaking a detailed study of social networks in Gorillas and Chimpanzees, a comprehensive research program of social complexity would provide a multi-faceted understanding of sociality. This would complement the social network analysis by exploring what makes managing social relationships cognitively complex, how group size and social structure affects the level of stress hormones, and how primates’ use of communication varies with group size.

### Social networks and group size in Gorillas

Among primates, larger groups are generally considered to exhibit greater social complexity than smaller ones, given the increased number of social relationships that must be maintained. Individuals in such groups are required to devote more time and effort to managing these relationships to sustain group cohesion and stability (Dunbar and Shultz 2007a; Manninen et al. 2017). Nevertheless, there is currently no standardized method for assessing social complexity across groups of varying sizes, and our understanding of how the patterning of social relationships shift with increasing group size remains limited (Dunbar and Shultz 2010). Gorilla groups vary greatly in size, with a range of 2–43 (Doran and McNeilage 2001; Robbins and Robbins 2018). Future research could collect data on a number of behavioural interactions (e.g. grooming, vocalisations, gestures, proximity, visual attention) in small, medium and large groups of Gorillas and carry out three main sets of network analyses. First, features of the overall network structure (e.g. connectedness, density) and the extent to which there are sub-structures within the overall network should be examined across the groups. Thus larger groups of Gorillas, especially those with more than one adult male, may be more likely to contain sub-groups. Network analysis is an ideal way of statistically identifying and characterizing such sub-groups, which are defined as nodes that are more densely connected to themselves than they are to other nodes in the network (Croft et al. 2008). Second, the extent to which the networks based on the different types of behavioural interactions overlap may be explored. There is a limit on the time available for grooming, so as group size increases, we predict that there will be an increasing dissociation between networks based on grooming and networks based on vocal and gestural communication, as Gorillas use communication rather than grooming to maintain their relationships. Third, use of network analysis would identify how age, sex and dominance rank affect the patterning of social relationships, and the roles that different individuals play in the group as a whole. Adult social bonds in Gorilla groups are strongest between females and silverbacks, with the females in the group forming weaker social bonds with each other (Doran and McNeilage 2001; Robbins and Robbins 2018). Network analysis allows precise quantification and statistical analysis of sex differences in the network characteristics and position of adult females and males. This type of data will lead to a comprehensive, quantitative understanding of the network structure of Gorillas groups, how Gorillas use different modes of interaction to manage their social relationships, the different roles the sexes play in Gorilla groups and how this changes with increasing group size.

### Social networks and group size in Chimpanzees

Chimpanzees live in a fission-fusion society, where individuals form socially and geographically circumscribed communities, within which they associate in temporary subgroups (parties) that vary in size, composition and duration (Lehmann and Boesch 2004; Mitani et al. 2002). Because of this dynamic and fluid social structure, discerning regularities in grouping, as well as spatiotemporal cohesion such as dispersal, range use and associations is more challenging for Chimpanzees than for primates that live in temporally and spatially stable groups such as Gorillas (Aureli and Schino 2019a). Thus the internal structuring of Chimpanzee communities, how this varies with group size and variations in sex differences in association patterns are all still poorly understood. Network analysis offers a powerful set of tools for characterising and analyzing individual associations within a population-level social context, and is particularly valuable in characterising complex fission-fusion social systems (Sueur et al. 2011). Chimpanzee community size can range from 20 to 150 (Lehmann and Boesch 2004), and it would be valuable to explore how social networks vary in small, medium and large communities of Chimpanzees. Particular attention may be given to identifying sub-structures within the wider community of Chimpanzees, as it is possible that the very large communities of Chimpanzees in fact consist of a number of sub-communities only loosely linked together. This has important implications for determining how many relationships an individual Chimpanzee has to keep track of, and thus how cognitive complexity increases as group size increases (Aureli and Schino 2019a). As with Gorillas, how the position and network characteristics of individual vary by age, sex and dominance rank may be explored. These individual-level characteristics influence the social complexity experienced by different animals in a group, which can vary widely between group members (Aureli and Schino 2019b). For example, in rhesus macaques, social networks reduce in size with age and thus on an individual level will have a smaller, less complex network to manage than younger macaques (Siracusa et al. 2022). Traditionally, male Chimpanzees have been seen as more gregarious than females, forming strong bonds with other males and distribute their activities more widely over their territories than females (Mitani et al. 2002). Thus male Chimpanzees would have a more complex social network than females, in that they have to maintain a greater variety of social ties than females, including larger numbers of weak ties which are cognitively demanding to keep track of (Dunbar 2024). Females, in contrast are often portrayed as less sociable, and spending most of their time with their own offspring, except when they are in oestrus. However, there is considerable variation in the extent of the sex differences in sociality in different populations of Chimpanzees (Lehmann and Boesch 2008). By exploring the extent to which position and network characteristics vary by sex, this can precisely identify the different roles male and female Chimpanzees play in the wider community, the extent of variation between individuals in these characteristics, and how this varies with group size. This would provide new network methods to analyse Chimpanzees’ complex sociality, and provide new insights into the cognitive challenges imposed by living in a fission-fusion system.

### Comparison of social networks in Gorillas and Chimpanzees

Chimpanzees and Gorillas are among our closest living relatives, and they exhibit remarkable diversity in various aspects of their social organisation both within and between species. Gorillas are folivores and their groups exhibit a low degree of fission-fusion dynamics in the membership of the group is stable temporally and spatially. In contrast Chimpanzees are frugivores with a high degree of fission-fusion dynamics. Thus a comparison of social structure in Chimpanzees and Gorillas provides an ideal opportunity to explore the implications of increasing group size for increased levels of social complexity, and how this is affected by the social organisation of the species. This would provide important insights into the nature and evolution of primate sociality. Comparisons between the two species can be made of the nature of the networks themselves, the extent to which the networks based on the different types of behavioural interactions overlap, the extent to which the groups or communities are based on a number of distinct sub-groups, and how the position and network characteristics of individuals vary by age, sex and dominance rank. Due to the differences in social organisation, an increase in group size in Gorillas results in them interacting with more individuals on a daily basis, whereas an increase in group size in Chimpanzees results in them having to manage more indirect relationships with individuals they may only see occasionally. Tracking these indirect relationships is hypothesised to be cognitively demanding, as in fission-fusion systems individuals must be able to retain and manipulate information about others (e.g. manipulating knowledge about third party relationships) whom they see only infrequently, as compared to systems with groups that are stable spatially and temporally where members see each other every day (Aureli and Schino 2019a; Barrett et al. 2003). By comparing two social networks in species with different forms of social organisation, and how these networks vary with group size, the cognitive demands of living in different social systems, and in groups of different sizes, can be determined. For example, how frequently do Chimpanzees actually encounter other members of the community, what sort of interactions do they have with these other individuals (grooming, proximity, vocal and gestural communication), how does this vary with group size and network structure, and how does this compare with Gorillas?

### Stress hormones, social networks and group size in Chimpanzees and Gorillas

A key part of examining social complexity is determining the extent to which increases in group size produces social stress for individual primates. Sociality can impose stress due to competition for resources such as food and mates, and thus living in large groups is predicted to be more demanding than living in smaller groups. Glucocorticoid (GC) is a hormone excreted in response to stress, and although in the short term, an increase in GC levels increases energy levels and can trigger behaviour which helps primates cope with environmental and social challenges, chronic stress can lead to reduced survival, fecundity and immunity (Abbott et al. 2003a, b). Glucocorticoid levels provide an objective way to estimate primates overall physiological well-being in different social circumstances, which can be used to complement measures based on behavioural data such as social affiliation patterns (Abbott et al. 2003a, b). One of the primary mechanisms to offset stress, both in humans and primates, is social affiliation (Dunbar 2010). GC levels in wild primates are sensitive to stressful events, such as the entry of a new male into the group, bringing a risk of infanticide (Crockford et al. 2008). Further, female baboons with a less diverse grooming network - meaning that they focused a greater proportion of their grooming effort on a smaller number of social partners - showed a faster decrease in levels of GC after the stressful event than females with a more diverse grooming network (Wittig et al. 2008).

This suggests how primates manage their social relationships can have a significant effect on their levels of stress, as measured by GC levels. However, it is currently not known how GC levels vary with group size in Chimpanzees or the Gorillas, with large groups predicted to be more stressful and thus resulting in higher GC levels. Further, individual variation in how primates adjust their social strategies in larger groups may affect their GC levels. For example, some individuals may adjust to an increase in group size by increasing their number of grooming partners, whereas others may actually reduce their number of grooming partners, and focus on their few key allies. Based on previous research (Crockford et al. 2008), it may be predicted that the latter strategy would be more effective in reducing stress, leading to lower GC levels. An important area of future research would be to examine how group size (small, medium and large groups of Gorillas and Chimpanzees), and individual variations in the pattern of social relationships, affects GC levels. This would give an objective, biological indicator of the social stress imposed by living in groups of different sizes, and thus provide important insights into the fitness consequences of sociality in primates (Abbott et al. 2003a, b).

### Social cognition, communication and social networks in Gorillas

One of the distinctive features of primate cognition is its flexibility, in that individuals can flexibly adjust their behaviour according to the current situation. This cognitive flexibility is required to monitor and manage social relationships in a dynamic social environment. Primates need to monitor both their own social bonds and the relationships between other group members, as shifts in third-party interactions—such as changes in dominance hierarchies—may influence their own standing within the group. There is a large body of evidence showing that primates have knowledge of third part relationships, in relation to, for example, mother-infant relationships, relative dominance rankings and matrilines (Silk 2007). In some contexts, primates may benefit from drawing on their understanding of both their own relationships and those among others to modify their behaviour based on the individuals present. For example, lower-ranking female Chimpanzees suppress their copulation calls if a high-ranking female is nearby to avoid female-female competition (Townsend et al. 2008). However, the extent to which Gorillas adjust their behaviour according to which other conspecifics are present - ‘audience effects’ - are not well understood. It is important to consider how these audience effects influence Gorillas’ vocal and gestural communication patterns in small, medium and large groups. For example, gestural communication may be used in situations where Gorillas do not want to broadcast a vocal signal to a wider audience. Further, the number of dyads and triads of social relationships that have to be socially managed increases as a power function of the number of individuals in a group (Dunbar and Shultz 2007b). Thus we can predict that it will become increasingly difficult for an individual to adjust their behaviour in groups of increasing size, and that Gorillas will therefore demonstrate less flexibility in communication patterns in larger groups. Finally, gestural communication in apes exhibits greater flexibility than vocal communication, and this study will explore the extent to which Gorillas are capable of using gestures and vocalisations flexibly according to the social situation. Examining this flexibility would provide insight into the cognitive complexity involved at the micro-level of managing social relationships, and how this varies with group size.

### Repertoire size and group size in Chimpanzees and Gorillas

Through hominin evolution there has been an increase in both brain size and this is likely to have been accompanied by an increase in group size (Aiello and Dunbar 1993). Dunbar (Dunbar 1993, 2012) has argued that the pressure to maintain larger social groups through hominin evolution may have driven the evolution of language as a novel social bonding mechanism that is more time efficient than grooming. Between primate species, it has been shown that evolutionary increases in the size of the vocal repertoire in non-human primates were associated with increases in both group size and also time spent grooming (McComb and Semple 2005). This suggests that vocal communication may indeed play a key role in the evolution of social behaviour - larger groups are more complex to manage, and thus require a larger repertoire to maintain an increasing number of differentiated relationships. However, it is increasingly being recognised that gestural communication also plays a key role in regulating social behaviour, and the role of gestural communication in wild primates in relation to sociality is still unclear (Byrne et al. 2017; Roberts and Roberts 2016). Future research could examine how both gestures and vocalisations in Chimpanzees and Gorillas are related to group size. There is currently an active debate as to whether human language evolved from vocal or gestural communication (Corballis 2009, 2017; McComb and Semple 2005), and how the usage and repertoire size of gestural and vocal communication varies with group size will provide important insights into this debate.

### Group size and culture in Chimpanzees and Gorillas

In human societies, culture is important in social bonding because it signals which social group one belongs to and promotes pro-social behaviour towards this group, in the absence of prior relationships or genetic relatedness (Van Schaik et al. 2012). In this context, social complexity is defined as the network where individuals interact with many unrelated individuals across many different social contexts, whilst cultural complexity is defined as systems which contain a larger number of distinct behavioural forms specific to social group. Socially complex societies thus possess more culturally complex features that differentiate it from other groups (Chick 1997). Whilst this perspective emphasizes the difference in cultural practises between the groups, the overlap in culture may also facilitate groups functioning together with other groups as a system of interdependent, complementary parts. Thus, both similarities and differences in culture define human social complexity.

In seeking to infer the evolution of culture in humans, a primary focus has been to understand culture in primates. Central to the study of culture in primates is the capacity for behavioural innovation and the transmission of these behaviours across individuals and generations through social learning. This transmission gives rise to behavioural patterns that tend to be interpreted similarly by members of the same cultural group (Van Schaik et al. 2012). The ethnographic approach, which identifies cultural traits by excluding ecological and genetic explanations for behavioural variation across populations, has yielded important insights into the evolutionary basis of culture (Whiten et al. 1999). Of particular interest is the capacity for culture in gestural and vocal communication. Within primates, cultural differences in gestural communication are well established and include grooming hand-clasp, leaf clipping, lip smacking, knuckle-knock, and heel-kick in Chimpanzees; the groom-slap and social scratch in bonobos; and chest beating, body slapping, ground slapping, and body touching in Gorillas (Badihi et al. 2023; Malherbe et al. 2025; McGrew et al. 2001; McGrew and Tutin 1978; Prieur et al. 2024; van Leeuwen et al. 2012, 2020; Watts 2016). Further, cultural differences in vocalisations such as alarm calls in orangutans and food grunts in the Chimpanzees has been claimed (Lameira et al. 2022; Watson et al. 2015). These studies demonstrated cultural differences in communication between the groups unaccounted for by environmental or genetic factors (Whiten et al. 1999). However, previous research has mostly considered single behaviour patterns or contexts and so far none of the research has systematically examined cultural differences in the morphology of single signals in the wild (Whiten 2017; Whiten et al. 1999). Hence, the extent to which animals possess communication dialects (culturally acquired differences in the form of the same signal type) is not well understood. Further, much of previous work has focused on behavioural variation, often excluding the role of culture in social bonding, on the assumption that the driving force is inheritance and adaptation, rather than conscious decision making (Whiten 2021).

The extent to which culture can act as a social bonding mechanism may be affected by the degree of overlap in the group-specific communicative repertoires between social partners. Unlike other forms of incidental similarity, culture serve as a particularly strong determinant of social bonds between unrelated individuals because they identify another’s goals and intentions as similar to one’s own. These shared goals create feeling of safety, because they are formed in normative contexts that prevent individuals from being harmed or exploited. Accordingly, social identities drive attention allocation in humans, whereby individuals allocate greater attention towards stimuli that are identity-consistent, while also shifting attention away from identity-inconsistent stimuli. This suggests that identities direct and influence decision-making, whereby individuals are motivated to perceive identity-consistent social environments (Coleman and Williams 2015).

Whilst extant research on primates has not examined the implications of culture on the allocation of attention, to safety, it has also largely overlooked the role of culture in the processing of social information. However, it is reasonable to suggest that communicative traditions function to enhance the relevance of social information and shape group-level dynamics by promoting the integration of social information in social encounters. Culture varies considerably in value in social bonding, suggesting its role in social differentiation and adjustment of social dynamics. In primates group-specific signals are more valuable than population-specific signals, whereas population -specific signals are more valuable than innate signals (e.g. facial expressions) because of their higher acquisition cost (Cohen 2012). In humans, the use of valuable, population-specific signals often corresponds to social identities that facilitate greater inclusivity toward unfamiliar individuals, enabling the formation of supportive friendships. Conversely, group specific signals of particularly high value within a group are typically associated with social hierarchies that promote or reinforce exclusivity (Van der Veen 2003). Differences in value would be demonstrated by in-group favouritism at out-group cost and divergence in group-specific signals (Nettle and Dunbar 1997). In contrast, out-group preferences at in-group cost would result in convergence in communication between groups. Describing and comparing the cultural diversity in communication across groups of Gorillas and the Chimpanzees which are genetically and ecologically homogenous but live in groups of different sizes would provide important insights into understanding of the evolution of social and cultural complexity.

### Social network analysis

Social network analyses is now established as key tool in behavioural analysis (Farine and Whitehead 2015; Krause et al. 2009; Sueur et al. 2011; Testard et al. 2022) (Kaburu et al. 2023). Social network analysis is important because it can take a number of different types of behavioural interactions e.g. grooming, vocalisations, gestures, proximity, body contact, visual attention, participation in coalitions, food sharing, social play and boundary patrols and directly compare them across dyads (Sueur et al. 2011). Further, multilayer networks can examine interdependencies between networks based on different behaviours (Hasenjager et al. 2021), whilst multiplex centrality can identify individuals who are well-connected across multiple network layers (Beisner et al. 2020; Vandeleest et al. 2025). The network analyses may be based on weighted, directed ties. The network is weighted in that the tie between two individuals, A and B, will be given a numerical value based on the rate or frequency of the behaviour. The network is directed in that the value of the tie from A to B may be different from that from B to A if there is inequality in the relationships (e.g. A grooms B more than B grooms A). If no interaction is observed in a particular category of interaction between a particular pair of individuals, the tie between those individuals will be scored as zero and undirected.

Once the value of the ties for all individuals in the network is known, different networks may be constructed for the different behavioural interactions listed above. However, computing the value of ties for all individuals in a network is often one of the biggest challenges in network analysis in wild animals and this can be affected by the sampling methods (Kaburu et al. 2023), so not always all ties can be used. Careful consideration needs to be given the sampling method to ensure the sampled network reflects the actual network, with scan samples effective at capturing many edges per scan as compared to focal sampling (Davis et al. 2018). The data analysis may then proceed through six steps for each of these networks (Krause et al. 2007). First, the information on social interactions may be organised into a matrix for data analysis, where the rows and columns represent individuals, and the values within the matrix represent the frequency of behavioural interaction. Second, the networks may be constructed and visualised. Algorithms such as ‘spring embedding’ may be used to arrange the network based on the rate of interactions (frequency per unit of time) between individuals, and thus reveal interesting network structural features. Arrows may be used to represent the directionality of social interactions, and thickness used to represent the weight of the tie. Attribute data (e.g. sex of individual) may also be incorporated into the network diagram. These diagrams can be a valuable way of seeing patterns in the networks, before proceeding onto the third step which is performing detailed network analysis (Sueur et al. 2011).

Network analysis provides a wealth of quantitative metrics that may be calculated to describe the social structure across different scales of organisation, from the individual to the population (Kaburu et al. 2023; Sueur et al. 2011). ‘Node-based’ measures may be used to examine the properties of how individual nodes are connected to each other in a network. Although many of these measures are based on binary networks, there are measures available for weighted and directed networks, (reviewed by Boccaletti et al. 2006). To give just two examples, node strength measures the total weight of all the ties connected to a node, and is thus the weighted equivalent of the binary measure node degree (the number of ties joined to a particular node). A weighted clustering coefficient may also be calculated, which measures the cliquishness of a network - the extent to which a nodes immediate neighbours that are themselves neighbours. These measures may be averaged over the network as a whole and be used to describe social organisation at the level of the group. The fourth step is to interpret these network measures, and the networks generated may be compared to randomized networks that provide a null model with which to test whether the observed network patterns are different from those expected by chance. For example, is the level of clustering observed in the network different from that which would be expected by chance? Weighted networks require different randomisation techniques than binary networks (Lusseau et al. 2008), and these type of methods may be used to examine if the observed networks are significantly different from chance.

Fifth, the network data may be used to look for non-random patterns of association between individuals (Croft et al. 2011; Farine and Carter 2022). A ‘community’ in a network is defined as a set of nodes that are more densely connected amongst themselves than they are to the rest of the network (Croft et al. 2008). Relating the communities found in networks to known individual characteristics, group characteristics or ecological variables can lead to a better understanding of the interplay between biological, ecological and other factors and the observed patterns of social interaction. These sub-structures would be difficult to detect using methods focused on the strength of bonds between dyads (e.g. Mitani 2009) or population methods, especially in fluid fission-fusion systems such as those found in Chimpanzees. Further, if a key property (e.g. node strength) varies significantly between communities, it is misleading to present a mean or medium value of that property over the whole population, as this ignores the internal structure of that population. Thus a key advance would be to identify these sub-structures within the groups of Chimpanzees and Gorillas, and examine how the number and properties of these sub-structures change with increasing group size. Again, although many of the statistical techniques used to detect communities in networks are based on binary networks, there are a small number of recently developed methods to detect communities in weighted networks and these types of methods may be used for community detection (Hajibabaei et al. 2023). Moving beyond dyad-based networks, simplicial complexes can be used to identify sub-grouping patterns. Simplicial sets can be used to represent interactions between more than two individuals, with simplicial complexes a specific type of simplicial set which contains all lower-order simplices i.e. also possible lower-order interactions (Iacopini et al. 2024).Finally, after quantifying the network and searching for sub-structures, the crucial step it to compare the observed network to other network. This may be done at three levels. First, networks based on the different behavioural interactions may be compared, to test the extent to which there is dissociation between, for example, the network based on the grooming data and the network based on the gesture data. These different interaction networks can also be combined into multiplex networks (Beisner et al. 2020)where inter-layer edges connect the same individuals in different layers (e.g. the grooming and gestures networks). Second, networks between the three different size groups *within* species may be compared, to explore how group size effects network structure within Gorillas and Chimpanzees. Finally, the networks may be compared *between* species, to explore the extent to which the differences in social organisation (fission-fusion vs. stable groups) and other differences between the species (e.g. in diet, in absolute group size) affect network structure. Comparing networks based on the same individuals is the most straightforward type of comparison, as the network has the same number of nodes and there are well-established statistical techniques for comparing these types of networks (Hemelrijk 1990). Comparing networks of different individuals is more problematic, as most network measures vary with the number of nodes and ties in the network. In this case, comparing key metrics across networks whilst controlling for differences in network size and structure can affect these metrics, can provide important insights into how networks vary within and across species (Albery et al. 2024). The majority of the methods developed to compare these types of networks are for binary, undirected networks. Methods for comparing weighted, directed networks are starting to be developed (Li et al. 2007), and a key part of future research will to be use these methods to compare weighted, directed networks (Kaburu et al. 2023). As many networks in both biological and social sciences are weighted and directed (even if they are often analysed as if they were binary) the set of results in respect to characterising, analysing and comparing weighted, directed networks would have wide applicability across a range of disciplines.

## Conclusions

A particularly challenging and unconventional aspect of the study of primate sociality lies in its use of social network analysis and in particular, use of weighted and directed ties to characterise the relationships between individuals. In weighted, directed networks a numerical value which reflects the strength of the tie, and there is the possibility of asymmetry in the ties. In contrast, the great majority of network analysis, in social sciences, biological sciences and mathematics, considers only binary networks, where the tie between two nodes is classified as present (1) or absent (0). This is appropriate for certain types of physical or mathematical networks and is often used as a simplifying assumption in the study of social networks. However, characterising a tie between two individuals in a binary fashion does not provide a rich insight into complex social relationships. Although it is clear when two animals are linked, in a binary network the difference between ties categorised by 1 is lost, and due to sampling issues it is rarely certain that two animals in a population are *not* linked, and thus the reliability of ties classed as 0 is often questionable. This severely limits the usefulness of network analysis in understanding *social* networks, where the weight and direction of the tie is a major component of the characterising the interaction between two individuals. Because analysing binary networks is more straightforward than analysing weighted networks, current approaches in social networks often use a cut-off value to transform weighted ties into binary ties. This is an unsatisfactory solution, as the cut-off is an arbitrary value, and where it is set can affect the resulting network structure (Lusseau et al. 2008). Whilst there has been some initial work on weighted, directed networks, the work is still in its infancy. If network analysis is to fulfil its potential in the study of *social* systems, it is necessary describe and compare weighted networks, so the nature of the tie between two individuals can be characterised more precisely.

This use of weighted ties is challenging, as techniques of analysing - and in particular comparing - weighted networks are less well established than those using binary networks, and work on weighted social networks in animals is in its infancy. However, the use of weighted networks, and the comparison between weighted networks of different sizes and in different species, has the potential to open up a major new field of research in network analysis, representing a major advance on the current reliance on binary network analysis. Given the inter-disciplinary nature of network analysis, this is likely to have wide applicability in many different fields of research, reaching across the mathematical, biological and social sciences.

Improving our understanding of primate social complexity is likely to lead to new insights into human evolution. Although much progress has been made in assessing the archaeological record, our understanding of hominin social life is in its infancy (Dunbar et al. 2014; Foley and Gamble 2009). Gorillas and Chimpanzees are two of our closest living ancestors, and as such an improved understanding of the forces governing their sociality will provide valuable insights into human social evolution. In particular, fission-fusion dynamics characterise Chimpanzee and bonobos (Furuichi 2009), and also are typical of modern-day hunter-gatherer (Aureli et al. 2008a, b; Marlowe 2005). This suggests that fission-fusion dynamics were characteristic of the social system of the last common ancestor of Chimpanzees, bonobos and modern humans (Aureli et al. 2008a, b; Foley and Gamble 2009). Further, a general trend in the course of human evolution is an increase in brain size, and this is likely to have been accompanied by a corresponding increase in social group size (Aiello and Dunbar 1993). A comparison of Gorillas and Chimpanzees offers the opportunity to explore the complexity involved in fission-fusion systems, as compared to more stable social groups, and how this complexity changes in groups of different sizes. This will help us understand how the social structure is likely to have changed with increasing group size in the fission-fusion system of early hominins, and the cognitive complexity involved in managing groups of increasing size (Aureli and Schino 2019a; Freeberg et al. 2012).

## Data Availability

No datasets were generated or analysed during the current study.
